# Safety of Gabapentin Prescribed for Any Indication in a Large Clinical Cohort of 571,718 US Veterans with and without Alcohol Use Disorder

**DOI:** 10.1111/acer.14408

**Published:** 2020-07-28

**Authors:** Christopher T. Rentsch, Kenneth L. Morford, David A. Fiellin, Kendall J. Bryant, Amy C. Justice, Janet P. Tate

**Affiliations:** ^1^ Faculty of Epidemiology & Population Health London School of Hygiene & Tropical Medicine London UK; ^2^ Veterans Aging Cohort Study Coordinating Center VA Connecticut Healthcare System West Haven Connecticut USA; ^3^ Department of Internal Medicine Yale School of Medicine New Haven Connecticut USA; ^4^ Center for Interdisciplinary Research on AIDS Yale School of Public Health New Haven Connecticut USA; ^5^ Director of HIV/AIDS Research National Institute on Alcohol Abuse and Alcoholism Bethesda Maryland USA

**Keywords:** Gabapentin, Neurologic Effects, Electronic Health Records, Alcohol Use Disorder, Chronic Hepatitis C, HIV Infection

## Abstract

**Background:**

Gabapentin is prescribed for seizures and pain and has efficacy for treating alcohol use disorder (AUD) starting at doses of 900 milligrams per day (mg/d). Recent evidence suggests safety concerns associated with gabapentin including adverse neurologic effects. Individuals with hepatitis C (HCV), HIV, or AUD may be at increased risk due to comorbidities and potential medication interactions.

**Methods:**

We identified patients prescribed gabapentin for ≥ 60 days for any indication between 2002 and 2015. We propensity‐score matched each gabapentin‐exposed patient with up to 5 gabapentin‐unexposed patients. We followed patients for 2 years or until diagnosed with (i) falls or fractures, or (ii) altered mental status using validated ICD‐9 diagnostic codes. We used Poisson regression to estimate incidence rates and relative risk (RR) of these adverse events in association with gabapentin exposure overall and stratified by age, race/ethnicity, sex, HCV, HIV, AUD, and dose.

**Results:**

Incidence of falls or fractures was 1.81 per 100 person‐years (PY) among 140,310 gabapentin‐exposed and 1.34/100 PY among 431,408 gabapentin‐unexposed patients (RR 1.35, 95% confidence interval [CI] 1.28 to 1.44). Incidence of altered mental status was 1.08/100 PY among exposed and 0.97/100 PY among unexposed patients, RR of 1.12 (95% CI 1.04 to 1.20). Excess risk of falls or fractures associated with gabapentin exposure was observed in all subgroups except patients with HCV, HIV, or AUD; however, these groups had elevated incidence regardless of exposure. There was a clear dose–response relationship for falls or fractures with highest risk observed among those prescribed ≥ 2,400 mg/d (RR 1.90, 95% CI 1.50 to 2.40). Patients were at increased risk for altered mental status at doses 600 to 2,399 mg/d; however, low number of events in the highest dose category limited power to detect a statistically significant association ≥ 2,400 mg/d.

**Conclusions:**

Gabapentin is associated with falls or fractures and altered mental status. Clinicians should be monitoring gabapentin safety, especially at doses ≥ 600 mg/d, in patients with and without AUD.

## Graphical abstract

Gabapentin is commonly prescribed and has shown potential utility in decreasing alcohol consumption among treatment‐seeking and non‐treatment‐seeking populations. We sought to determine its association with neurologic events (falls or fractures and altered mental status) among patients receiving gabapentin for any indication. In this national study of over 500,000 patients, gabapentin‐exposed patients had increased incidence of neurologic adverse events compared to unexposed patients. Clinicians should be monitoring gabapentin safety, especially at doses ≥600 mg/day.

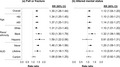

The limited efficacy of current medication and counseling strategies to treat alcohol use disorder (AUD) has led to a search for new medications, especially ones with which prescribers are comfortable. Gabapentin, an anticonvulsant and structural analogue to gamma‐aminobutyric acid (GABA), is approved by the U.S. Food and Drug Administration for treating partial seizure and postherpetic neuralgia and has shown efficacy for the treatment of AUD (Mason et al., 2014; Pani et al., 2014). Gabapentin is also commonly prescribed off‐label for peripheral neuropathy, fibromyalgia, and other painful conditions (Kesselheim et al., 2011; Shanthanna et al., 2017).

Recent evidence suggests that clinicians are comfortable and familiar with gabapentin. A qualitative analysis examining attitudes toward gabapentin use for managing neuropathic pain found that prescribers viewed gabapentin to be a safe and nonaddictive medication with few drug interactions (Ghinea et al., 2015). In 2016, 64 million gabapentin prescriptions were dispensed making it the 10^th^ most prescribed medication in the United States (IQVIA Institute, 2018). Prescribing rates of gabapentin have increased considerably in recent years partially due to promotion of gabapentin for on‐ and off‐label uses (Steinman et al., 2006) as well as the perception that it represents a safe alternative to opioids for treating chronic pain (Goodman and Brett, 2017).

However, gabapentin presents important safety concerns including neurologic adverse effects, such as ataxia, dizziness, and somnolence (Meng et al., 2014; Shanthanna et al., 2017; Wiffen et al., 2017). This is of particular concern for populations who may be at increased risk for adverse effects, such as individuals with hepatitis C (HCV), HIV, and AUD. These patients may be at greater risk of neurologic adverse events due to medical and psychiatric comorbidities and potential medication interactions. As alterations in mental status and coordination can lead to falls, fractures are a particular concern due to the association of HIV infection with fragility fractures (Womack et al., 2011) and AUD with osteoporosis and osteopenia (Berg et al., 2008).

Given the widespread prescribing of gabapentin and its potential utility in decreasing alcohol consumption among treatment‐seeking and non–treatment‐seeking populations (Rentsch et al., 2019), we sought to determine its association with events often linked with neurologic effects of dizziness, ataxia, and somnolence among patients receiving gabapentin for any indication, specifically falls or fractures (Ishida et al., 2018) and altered mental status. We further assessed whether these effects differed in demographic and clinical subpopulations at higher risk for these events, including patients with and without HCV, HIV, and AUD.

## Materials and Methods

### Study Population

We used electronic health record (EHR) data available through the US Department of Veterans Affairs (VA) national Corporate Data Warehouse (CDW). The VA is the largest integrated healthcare system in the United States and comprises over 800 community outpatient clinics, 150 hospitals and medical centers, and 120 nursing homes. We extracted data on all patients born between 1945 and 1965 who had at least 1 outpatient visit on or after 1 October 1999, which included approximately half of all Veterans in care. This study has been approved by the institutional review boards of the VA Connecticut Healthcare System and Yale School of Medicine, granted a waiver of informed consent, and deemed Health Insurance Portability and Accountability Act compliant.

We included patients who did (gabapentin exposed) and did not (gabapentin unexposed) receive gabapentin dispensed at VA pharmacies. We did not consider other gabapentinoids (e.g., pregabalin or gabapentin enacarbil) in this analysis as they are not commonly prescribed in the VA. The gabapentin‐exposed group included all patients who received 2 or more gabapentin fills for at least 60 continuous days, for any indication, between January 1, 2002, and March 30, 2015, from the following VA clinics: primary care, mental health, neurology, general internal medicine, physical medicine and rehabilitation services, pain, podiatry, orthopedics, women’s clinic, psychiatry, substance use, and rheumatology. These clinics were chosen because they were the source of most gabapentin prescriptions. To ensure that unexposed patients came from the same source population and had an equal opportunity to receive gabapentin, we randomly selected one outpatient visit date per calendar year to identify patients who attended one of the listed clinics but never received gabapentin.

To allow us to follow exposed and unexposed patients over similar calendar time, we created an “index date” (also referred to as “baseline”), which was defined as the first fill date for gabapentin‐exposed patients and the random outpatient visit date for unexposed patients. We identified the first prescription for gabapentin during the study period and required a 180‐day washout period so as to identify new episodes of gabapentin exposure. We excluded patients with no outpatient care in the year prior to their index date, because of unknown recent medical history.

### Propensity Score Model and Matching

In clinical trials, randomization is used to balance the distribution of all potential confounders across treatment groups. To emulate randomization using observational data, we used propensity score matching. This was done by first modeling the probability (i.e., propensity) of receiving the treatment of interest as a function of all measured covariates (Brookhart et al., 2006). Exposed patients were then matched to unexposed patients with a similar propensity. Matching by propensity score creates balanced exposure groups similar to treatment allocation in a randomized controlled trial (Austin, 2011), thus addressing concerns of confounding by indication. Unexposed patients with very low propensity and exposed patients with very high propensity are unlikely to match, which is akin to inclusion and exclusion criteria of a trial.

**Fig. 1:**
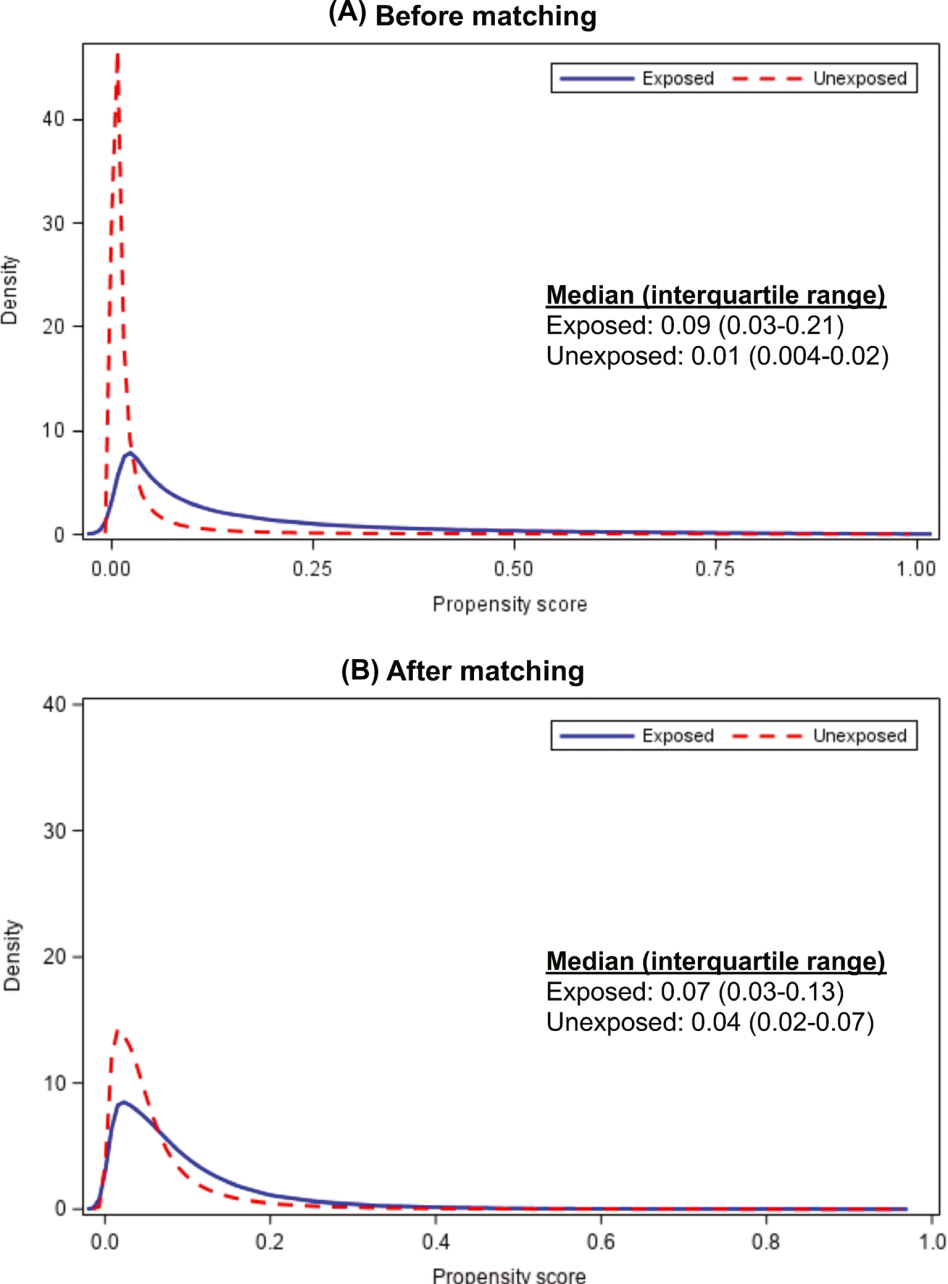
Distribution of propensity scores of gabapentin exposure before and after propensity score matching. Panel (**A**): Before matching. Panel (**B**): After matching

In our study, propensity scores were used to account for the probability of being prescribed gabapentin given a set of covariates that are associated with both gabapentin receipt and neurologic adverse events or associated only with neurologic adverse events. Propensity scores (i.e., the predicted probability of gabapentin exposure) were estimated using a multivariable logistic regression model. Variables used in the propensity score models were selected a priori based on clinical knowledge (Hernan et al., 2002) and included the following: year of index date, age at baseline, race/ethnicity, smoking status, body mass index at baseline, site prescribing pattern (the proportion of patients who initiated gabapentin stratified by year), laboratory values closest to the index date (including hemoglobin, international normalized ratio, triglycerides), HCV status, HIV status, history of seizure prior to baseline, diabetes complications severity index (Young et al., 2008) at baseline, history of pain diagnoses prior to baseline (including neuropathy, osteoarthritis, or pain in the abdomen, back, chest, extremity, or neck, headache, or fracture), and history of medical and psychiatric conditions prior to baseline (including atrial fibrillation, myocardial infarction/coronary artery disease, peripheral vascular disease, diabetes, nephrolithiasis, glomerulonephritis, hyperlipidemia, pancreatitis, drug use disorders, posttraumatic stress disorder (PTSD), major or other depression, anxiety, bipolar disorder, schizophrenia, and schizoaffective disorder). We also included variables that captured attendance to clinics (including primary care, dialysis, diabetic retinal screening, rheumatology, infectious disease, nephrology, neurology, pain, allergy, chiropractic, dental, diabetes, emergency department, electrocardiogram laboratory, ophthalmology, hematology, oncology, homeless program, nutrition, orthopedics, substance use, mental health, PTSD), frequency of all‐cause hospitalizations, and the total number of unique clinics visited in the year prior to baseline. Lastly, variables denoting receipt of other prescriptions at baseline to treat pain (including benzodiazepines, nonsteroidal anti‐inflammatory drugs (NSAIDs), opioids, muscle relaxants, and antidepressants) and seizures were included in the model. Interaction terms were explored for significance, and 5 were kept in the final model (all *p* < 0.05). The model c‐statistic was 0.89 indicating adequate discrimination between gabapentin‐exposed and gabapentin‐unexposed patients (Hosmer and Lemeshow, 2000). Since the distribution of propensity scores for exposed patients was different than that of unexposed patients, we used propensity score matching to exclude nonexchangeable unexposed patients (i.e., those with extremely low probability of gabapentin exposure) (Fig. 1) (Spoendlin et al., 2016). Each exposed patient was matched to up to 5 unexposed patients in the same calendar year, using a greedy matching algorithm (Cormen, 2009).

### Clinical Subpopulations

For all conditions, we required 1 inpatient or 2 outpatient diagnostic codes using the International Classification of Diseases, 9^th^ revision (ICD‐9). HCV infection was defined by any confirmatory HCV RNA test or ICD‐9 diagnostic codes 070.41/0.44; 070.51/0.54; 070.70/0.71; or V02.62. HIV status was determined by ICD‐9 diagnostic codes 042, 044, or V08. AUD status was determined by ICD‐9 diagnostic codes 303.X or 305 to 305.03 at any time prior to baseline. The date of AUD diagnosis was used to categorize AUD status into 3 mutually exclusive categories: never, lifetime (before the year prior to the index date), or current (1 year prior to or 180 days after the index date). Patients with both lifetime and current AUD diagnoses were classified as current.

### Neurologic Adverse Events and Follow‐Up

We used ICD‐9 codes to define falls or fractures (805.2X‐805.7, 812.XX, 820.XX, E882‐E885, E888) and altered mental status (291, 291.1, 292.81, 293, 293.1, 298.2, 780.09, 780.97). These ICD‐9 codes were selected to be consistent with previous literature (Hope et al., 2014; Pugh et al., 2015; Thyagarajan et al., 2013; Womack et al., 2011). We excluded patients diagnosed with any of these events in the year preceding their index date.

Patients were followed a maximum of 2 years from their index date to the first occurrence of any outcome, last VA visit, death, or September 30, 2015. Additionally, gabapentin‐exposed patients were censored 30 days after the end of their last gabapentin prescription (allowing for a maximum 30‐day gap between fills). To ensure equal follow‐up time within matched sets, unexposed patients were censored at the total follow‐up time of their matched exposed patient.

### Statistical Analyses

We used standardized differences (Austin, 2009) to examine balance between exposed and unexposed patients included in the full and propensity‐score matched sample. We estimated incidence rates (IR) for exposed and unexposed patients for each outcome. We then used multivariable Poisson regression models to estimate exposure rate ratios (RR) and 95% confidence intervals (CI) for the relative risk of a neurologic adverse event in association with exposure to gabapentin. We performed subgroup analyses by age (<60 or ≥ 60 years), race/ethnicity (white, black, or Hispanic), sex (male or female), HCV status, HIV status, and AUD (never, lifetime, or current). Finally, we investigated association of initial gabapentin dose with neurologic adverse events (<600 mg, 600 to 899 mg, 900 to 1,199 mg, 1,200 to 1,799 mg, 1,800 to 2,399 mg, and ≥ 2,400 mg). All statistical analyses were performed using SAS version 9.4 (SAS Institute Inc., Cary, NC).

## Results

### Sample

We identified 431,920 gabapentin‐exposed patients and 2,576,410 gabapentin‐unexposed patients eligible for propensity score matching. The matching process resulted in 54,878 (39.1%) gabapentin‐exposed patients with 5 unexposed matches, 8,475 (6.0%) with 412,577 (9.0%) with 321,007 (15.0%) with 2, and 43,373 (30.9%) with 1 matched unexposed patient. Thus, the analytic sample consisted of 140,310 exposed and 431,408 unexposed patients.

Before propensity score matching, the distribution of baseline characteristics significantly differed between gabapentin‐exposed and gabapentin‐unexposed patients (Table 1). Compared to gabapentin‐exposed patients who matched to unexposed patients, gabapentin‐exposed patients who did not match had similar proportions with HCV, HIV, or current AUD, were more likely to have comorbidities, particularly neuropathic pain (28%) and be prescribed other medications particularly opioids (30%) and muscle relaxants (18%) (Table S1). The median (interquartile range) propensity score in exposed patients who did not match was 0.11 (0.03 to 0.27), higher than those who were matched 0.07 (0.03 to 0.13). In the matched sample, gabapentin‐exposed and gabapentin‐unexposed patients were well balanced. Median follow‐up time was 137 days (IQR 100 to 269 days). Among exposed patients in the analytic sample, 38%, 20%, 28%, 7%, 5%, and 3% were initially prescribed daily doses of gabapentin <600 mg, 600 to 899 mg, 900 to 1,199 mg, 1,200 to 1,799 mg, 1,800 to 2,399 mg, and ≥2,400 mg, respectively.

**Table 1:** Distribution of Baseline Characteristics in Gabapentin‐Exposed and Gabapentin‐Unexposed Patients Before and After Propensity Score (PS) Matching Among Those Meeting Model Inclusion Criteria

Characteristic	Full cohort		Std. diff	PS‐matched		Std. diff
	Exposed	Unexposed		Exposed	Unexposed	
	*n* = 431,920	*n* = 2,576,410^a^		*n* = 140,310	*n* = 140,310^ a^	
Age						
<60	267,328 (61.9)	1,572,375 (61.0)	<0.01	88,980 (63.4)	87,641 (62.5)	0.02
≥60	164,592 (38.1)	1,004,035 (39.0)		51,330 (36.6)	52,669 (37.5)	
Race/ethnicity						
White	302,615 (70.1)	1,631,928 (63.3)	0.16	97,513 (69.5)	95,534 (68.1)	0.07
Black	76,133 (17.6)	454,832 (17.7)		24,815 (17.7)	25,382 (18.1)	
Hispanic	23,167 (5.4)	117,604 (4.6)		7,512 (5.4)	7,332 (5.2)	
Other	13,518 (3.1)	75,734 (2.9)		4,454 (3.2)	4,194 (3.0)	
Missing	16,487 (3.8)	296,312 (11.5)		6,016 (4.3)	7,867 (5.6)	
Male sex	401,937 (93.1)	2,425,510 (94.1)	0.03	131,007 (93.4)	131,835 (94.0)	0.02
HCV+	39,864 (9.2)	115,976 (4.5)	0.14	12,384 (8.8)	13,271 (9.5)	0.01
HIV+	4,193 (1.0)	18,257 (0.7)	0.03	1,381 (1.0)	1,616 (1.2)	0.01
AUD						
Never	337,384 (78.1)	2,290,858 (88.9)	0.20	111,937 (79.8)	108,671 (77.5)	0.02
Lifetime	43,411 (10.1)	140,080 (5.4)		13,336 (9.5)	14,471 (10.3)	
Current	51,125 (11.8)	145,472 (5.7)		15,037 (10.7)	17,169 (12.2)	
Any hospitalization	68,054 (15.8)	160,016 (6.2)	0.29	20,494 (14.6)	22,748 (16.2)	0.03
Conditions						
Seizure	10,885 (2.5)	32,187 (1.3)	0.10	3,166 (2.3)	3,960 (2.8)	<0.01
Diabetes	165,085 (38.2)	395,961 (15.4)	0.42	51,017 (36.4)	50,963 (36.3)	0.09
Anxiety	82,884 (19.2)	184,599 (7.2)	0.27	24,277 (17.3)	23,186 (16.5)	0.07
Neuropathic pain	111,164 (25.7)	95,761 (3.7)	0.59	28,524 (20.3)	25,754 (18.4)	0.19
Any chronic pain	401,994 (93.1)	1,751,616 (68.0)	0.41	129,072 (92.0)	128,342 (91.5)	0.08
Other prescription						
Benzodiazepines	61,015 (14.1)	92,721 (3.6)	0.32	17,786 (12.7)	17,950 (12.8)	0.09
Opioid	119,878 (27.8)	121,218 (4.7)	0.62	32,163 (22.9)	31,987 (22.8)	0.16
Antidepressant	51,802 (12.0)	53,710 (2.1)	0.36	13,697 (9.8)	12,783 (9.1)	0.12
NSAID	207,325 (48.0)	374,108 (14.5)	0.66	62,935 (44.9)	64,879 (46.2)	0.11
Muscle relaxant	69,626 (16.1)	60,830 (2.4)	0.46	17,755 (12.7)	17,220 (12.3)	0.13
Anticonvulsant	12,387 (2.9)	22,179 (0.9)	0.14	3,631 (2.6)	3,665 (2.6)	0.04

PS, propensity score; HIV, human immunodeficiency virus; HCV, hepatitis C virus; AUD, alcohol use disorder; NSAID, nonsteroidal anti‐inflammatory drug.

^a^Unexposed matches were weighted according to number of matches.

All statistics reported as *n* (%); up to 5 unexposed patients were matched to each exposed patient.

### Rates of Adverse Events

**Fig. 2:**
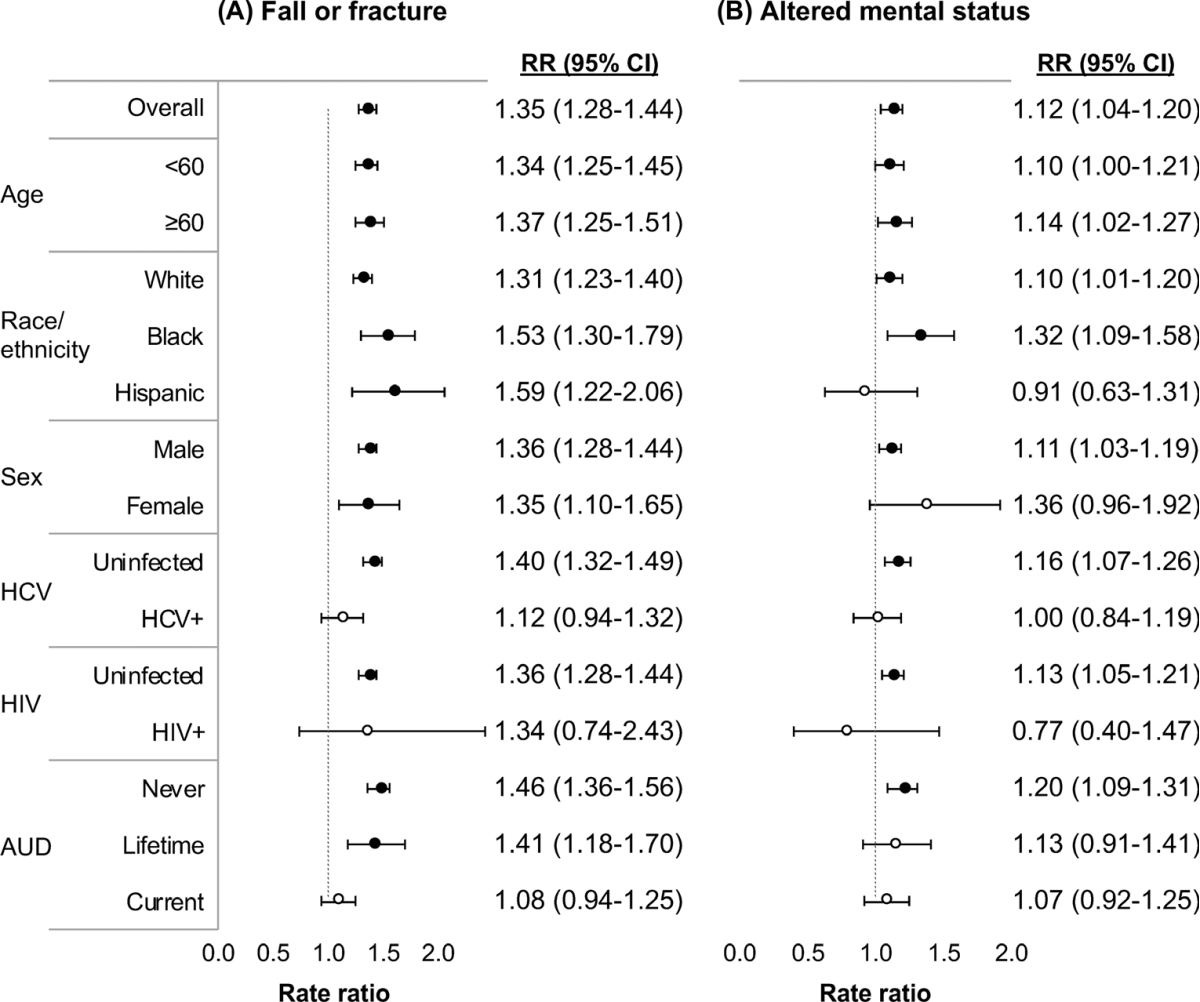
Associations between gabapentin exposure and neurologic adverse events. Panel (**A**): Fall or fracture. Panel (**B**): Altered mental status. *Note:* ● = *p* ≤ 0.05; ○ = *p* > 0.05. *Abbreviations:* RR, rate ratio; CI, confidence interval; HCV, hepatitis C virus; HIV, human immunodeficiency virus; AUD, alcohol use disorder.

Overall in the matched sample, the incidence rate for falls or fractures was 1.81 per 100 person‐years (PY) among exposed and 1.34/100 PY among unexposed patients, and for altered mental status 1.08/100 PY among exposed and 0.97/100 PY among unexposed patients (Table 2). Compared to unexposed patients, those exposed to gabapentin were 35% more likely (95% confidence interval [CI] 1.28 to 1.44) to experience a fall or fracture and 12% more likely (95% CI 1.04 to 1.20) to experience altered mental status (Fig. 2).

**Table 2:** Rates of Neurologic Adverse Events Among 140,310 Gabapentin‐Exposed Patients and 1:5 Propensity‐Score Matched Unexposed Controls

	Exposed	Fall or fracture				Altered mental status			
		# Events	Rate (95% CI)	RR (95% CI)	*p*‐value	# Events	Rate (95% CI)	RR (95% CI)	*p*‐value
Overall	Yes	1,663	1.81 (1.73 to 1.90)	1.35 (1.28 to 1.44)	<0.0001	1,001	1.08 (1.02 to 1.15)	1.12 (1.04 to 1.20)	0.0027
	No	3,547	1.34 (1.29 to 1.38)	ref		2,600	0.97 (0.93 to 1.01)	ref	
Age, years									
<60	Yes	992	1.79 (1.69 to 1.91)	1.34 (1.25 to 1.45)	<0.0001	558	1.00 (0.92 to 1.09)	1.10 (1.00 to 1.21)	0.0578
	No	2,162	1.34 (1.28 to 1.39)	ref		1,488	0.91 (0.87 to 0.96)	ref	
≥60	Yes	671	1.83 (1.70 to 1.98)	1.37 (1.25 to 1.51)	<0.0001	443	1.21 (1.10 to 1.32)	1.14 (1.02 to 1.27)	0.0193
	No	1,385	1.34 (1.27 to 1.41)	ref		1,112	1.06 (1.00 to 1.12)	ref	
Race/ethnicity									
White	Yes	1,276	1.88 (1.78 to 1.99)	1.31 (1.23 to 1.40)	<0.0001	743	1.09 (1.01 to 1.17)	1.10 (1.01 to 1.20)	0.0249
	No	2,588	1.43 (1.38 to 1.49)	ref		1,798	0.99 (0.94 to 1.04)	ref	
Black	Yes	209	1.60 (1.40 to 1.83)	1.53 (1.30 to 1.79)	<0.0001	151	1.15 (0.98 to 1.35)	1.32 (1.09 to 1.58)	0.0036
	No	522	1.05 (0.96 to 1.14)	ref		449	0.88 (0.80 to 0.96)	ref	
Hispanic	Yes	84	1.92 (1.55 to 2.38)	1.59 (1.22 to 2.06)	0.0005	37	0.84 (0.61 to 1.16)	0.91 (0.63 to 1.31)	0.6220
	No	173	1.21 (1.04 to 1.41)	ref		134	0.92 (0.78 to 1.09)	ref	
Sex									
Male	Yes	1,528	1.77 (1.69 to 1.86)	1.36 (1.28 to 1.44)	<0.0001	954	1.10 (1.03 to 1.17)	1.11 (1.03 to 1.19)	0.0069
	No	3,259	1.31 (1.26 to 1.35)	ref		2,500	0.99 (0.96 to 1.03)	ref	
Female	Yes	135	2.38 (2.01 to 2.82)	1.35 (1.10 to 1.65)	0.0041	47	0.82 (0.62 to 1.10)	1.36 (0.96 to 1.92)	0.0817
	No	288	1.77 (1.58 to 1.98)	ref		100	0.61 (0.50 to 0.74)	ref	
HCV infection									
Uninfected	Yes	1,478	1.74 (1.65 to 1.83)	1.40 (1.32 to 1.49)	<0.0001	832	0.97 (0.91 to 1.04)	1.16 (1.07 to 1.26)	0.0003
	No	3,028	1.24 (1.20 to 1.29)	ref		2,069	0.84 (0.80 to 0.88)	ref	
HCV+	Yes	185	2.71 (2.34 to 3.12)	1.12 (0.94 to 1.32)	0.1956	169	2.47 (2.12 to 2.87)	1.00 (0.84 to 1.19)	0.9865
	No	519	2.42 (2.22 to 2.64)	ref		531	2.46 (2.26 to 2.68)	ref	
HIV infection									
Uninfected	Yes	1,648	1.81 (1.72 to 1.90)	1.36 (1.28 to 1.44)	<0.0001	990	1.08 (1.02 to 1.15)	1.13 (1.05 to 1.21)	0.0015
	No	3,507	1.33 (1.29 to 1.38)	ref		2,548	0.96 (0.92 to 1.00)	ref	
HIV+	Yes	15	2.00 (1.21 to 3.32)	1.34 (0.74 to 2.43)	0.3333	11	1.46 (0.81 to 2.64)	0.77 (0.40 to 1.47)	0.4210
	No	40	1.50 (1.10 to 2.04)	ref		52	1.91 (1.45 to 2.51)	ref	
AUD									
Never	Yes	1,263	1.68 (1.59 to 1.77)	1.46 (1.36 to 1.56)	<0.0001	680	0.90 (0.83 to 0.97)	1.20 (1.09 to 1.31)	<0.0001
	No	2,418	1.15 (1.10 to 1.19)	ref		1,598	0.75 (0.71 to 0.79)	ref	
Lifetime	Yes	166	2.02 (1.73 to 2.35)	1.41 (1.18 to 1.70)	0.0002	108	1.31 (1.08 to 1.58)	1.13 (0.91 to 1.41)	0.2803
	No	371	1.43 (1.29 to 1.58)	ref		307	1.16 (1.04 to 1.30)	ref	
Current	Yes	234	2.84 (2.49 to 3.22)	1.08 (0.94 to 1.25)	0.2869	213	2.57 (2.25 to 2.94)	1.07 (0.92 to 1.25)	0.3661
	No	758	2.62 (2.44 to 2.81)	ref		695	2.40 (2.23 to 2.58)	ref	

AUD, alcohol use disorder; CI, confidence interval; HIV, human immunodeficiency virus; HCV, hepatitis C virus; RR, rate ratio.

Rates per 100 person‐years.

### Subgroup Analyses

Compared to the overall sample, incidence rates for both neurologic adverse events were greater among those with HCV infection, HIV infection, or current AUD diagnosis. Although absolute incidence was higher, the relative risk associated with gabapentin exposure was attenuated (Table 2). The rate of falls or fractures for patients with HCV infection was 2.71/100 PY among exposed and 2.42/100 PY among unexposed (rate ratio [RR] 1.12, 95% CI 0.94 to 1.32). For patients with HIV infection, rates were 2.00/100 PY among exposed and 1.50/100 PY among unexposed (RR 1.34, 95% CI 0.74 to 2.43). For patients with current AUD diagnosis, rates were 2.84/100 PY among exposed and 2.62/100 PY among unexposed (RR 1.08, 95% CI 0.94 to 1.25). In all other subgroups, excess risk associated with gabapentin exposure persisted (Fig. 2). Similar results were observed for altered mental status in the various subgroups.

We further stratified exposed patients by dose of gabapentin. There was a clear dose–response relationship for falls or fractures with relative risks increasing from 1.23 (95% CI 1.13 to 1.34) for <600 mg/d to 1.90 (95% CI 1.50 to 2.40) for ≥ 2,400 mg/d. (Table 3). Patients were at elevated risk for altered mental status at doses ≥ 600 mg/d; however, associations in dose categories ≥ 1,800 mg/d were not statistically significant, possibly due to few events in these higher dose categories.

**Table 3:** Dose‐Specific Rates of Neurologic Adverse Events Among 140,310 Gabapentin‐Exposed Patients and 1:5 Propensity‐Score Matched Unexposed Controls

	Fall or fracture				Altered mental status			
	# Events	Rate (95% CI)	RR (95% CI)	*p*‐value	# Events	Rate (95% CI)	RR (95% CI)	*p*‐value
Dose, milligrams								
≥2,400	71	2.56 (2.03 to 3.23)	1.90 (1.50 to 2.40)	<0.0001	33	1.19 (0.84 to 1.67)	1.21 (0.86 to 1.71)	0.2724
1,800 to 2,399	92	1.92 (1.57 to 2.36)	1.43 (1.16 to 1.76)	0.0008	60	1.25 (0.97 to 1.61)	1.28 (0.99 to 1.65)	0.0597
1,200 to 1,799	124	2.05 (1.72 to 2.45)	1.52 (1.27 to 1.82)	<0.0001	80	1.32 (1.06 to 1.65)	1.35 (1.08 to 1.69)	0.0082
900 to 1,199	466	1.98 (1.80 to 2.16)	1.47 (1.33 to 1.62)	<0.0001	294	1.25 (1.11 to 1.40)	1.27 (1.13 to 1.43)	<0.0001
600 to 899	320	1.76 (1.58 to 1.96)	1.31 (1.17 to 1.47)	<0.0001	215	1.18 (1.03 to 1.35)	1.21 (1.05 to 1.39)	0.0082
<600	590	1.66 (1.53 to 1.79)	1.23 (1.13 to 1.34)	<0.0001	319	0.90 (0.80 to 1.00)	0.91 (0.81 to 1.03)	0.1260
Unexposed	3,545	1.35 (1.30 to 1.39)	Ref		2,580	0.98 (0.94 to 1.02)	Ref	

RR, rate ratio; CI, confidence interval.

Rates per 100 person‐years.

## Discussion

In this national study of over 500,000 patients aged between 36 and 70 years, gabapentin‐exposed patients had increased incidence of neurologic adverse events compared to unexposed patients and were 35% more likely to experience a fall or fracture and 12% more likely to experience altered mental status. Patients with HCV infection, HIV infection, or a current AUD diagnosis had elevated rates of all neurologic adverse events compared to overall rates, but did not demonstrate increased risk of neurologic events when exposed to gabapentin. Our findings also showed a positive dose–response relationship for falls or fractures at all doses with greatest risk of occurring at gabapentin doses ≥ 2,400 mg/d and increased risk of altered mental status at doses ≥ 600 mg/d.

Our overall finding that gabapentin‐exposed patients were more likely to experience neurologic adverse events is consistent with several studies. A systematic review and meta‐analysis evaluating the safety of gabapentinoids in chronic low back pain found that patients receiving gabapentin were significantly more likely to report dizziness, fatigue, and difficulties with mentation compared to placebo (Shanthanna et al., 2017). Similarly, a Cochrane Collaborative review of 37 randomized controlled trials examining gabapentin for chronic neuropathic pain found that adults taking gabapentin experienced significantly more gait disturbance, dizziness, and somnolence compared to those receiving placebo (Wiffen et al., 2017). A meta‐analysis of 7 randomized controlled trials involving a total of 2,039 patients found that patients given gabapentin for the treatment of postherpetic neuralgia were significantly more likely to experience ataxia, dizziness, and somnolence (Meng et al., 2014). Another meta‐analysis of 7 randomized controlled trials assessing safety and efficacy of different doses of gabapentin for postherpetic neuralgia found that gabapentin at 1,800 mg/d was significantly associated with dizziness and somnolence and the risk of these adverse events increased at doses of 2,400 to 3,600 mg/d (Wang and Zhu, 2017). Unlike these studies that examined adverse effects such as dizziness and ataxia, we focused on falls and fractures, which are less reported in the literature. One large observational study among 140,899 Medicare‐covered adults receiving hemodialysis found that gabapentin was associated with 55% increased risk of falls and 38% increased risk of fractures (Ishida et al., 2018).

Despite evidence demonstrating gabapentin’s neurologic adverse events especially when used for pain, its use for treating individuals with AUD is generally considered safe. One randomized controlled trial evaluating the impact of gabapentin dose on alcohol‐related outcomes among 150 patients with current AUD reported no significant differences in the number, severity, or type of adverse events between patients receiving placebo, gabapentin 900 mg/d, or gabapentin 1,800 mg/d, although 4 of the 5 identified drug‐related adverse events occurred in gabapentin‐exposed patients (fatigue and headache) (Mason et al., 2014). A meta‐analysis of 7 placebo‐controlled randomized controlled trials evaluating efficacy of gabapentin for treating AUD also found no serious adverse events reported with gabapentin exposure (Kranzler et al., 2019). Similarly, we found less than 10% excess risk (not statistically significant) of adverse events associated with gabapentin exposure among patients with current AUD. However, a recent randomized controlled trial examining the efficacy of gabapentin on AUD treatment outcomes among 90 patients with alcohol withdrawal symptoms found significantly more reports of mild to moderate dizziness in those receiving gabapentin versus placebo (*n* = 25 vs. *n* = 15; *p* = 0.02) (Anton et al., 2020). Additionally, a multisite clinical trial evaluating the safety and efficacy of gabapentin enacarbil extended‐release (GE‐XR) in 346 participants with moderate to severe AUD reported no serious adverse events related to medication use, but did find significantly greater rates of fatigue (25.9% vs. 15.5%; *p* = 0.022), somnolence (17.6% vs 9.5%; *p* = 0.038), and tremor (5.9% vs. 0.6%; *p* = 0.010), as well as a nonsignificant increase for dizziness (21.2% vs. 13.7%; *p* = 0.085) in the GE‐XR group versus placebo group (Falk et al., 2019).

Compared to overall rates, we found that patients with current AUD had greater rates of neurologic adverse events regardless of gabapentin exposure, suggesting that these patients represent a particularly vulnerable population at risk for falls, fractures, and altered mental status. Given the growing body of evidence supporting gabapentin’s safety and efficacy in treating AUD (Anton et al., 2020; Kranzler et al., 2019; Mason et al., 2014), these baseline risks among individuals with current AUD should be considered alongside the potential benefits gabapentin treatment in this population. Among trials that found significantly greater rates of adverse events among gabapentin‐exposed patients, these events were reported as mild to moderate (Anton et al., 2020; Falk et al., 2019; Pani et al., 2014). Furthermore, Anton and colleagues (2020) reported significantly more dizziness in those receiving gabapentin versus placebo, but the presence or absence of dizziness did not significantly account for gabapentin’s effectiveness. Importantly, these clinical trials included only medically stable patients who were not using substances other than alcohol and nicotine, and thus may not reflect our study sample or generalize to other treatment settings.

Similar to patients with current AUD, our subgroup analysis of patients with HCV and HIV demonstrated elevated incidence of adverse events regardless of gabapentin exposure, which may reflect baseline risk for these adverse outcomes due to increased rates of medical and psychiatric comorbidities and potential medication interactions (Evon et al., 2018; Ruzicka et al., 2019). In contrast to our findings, one small randomized controlled trial comparing the effect of gabapentin with placebo in HIV‐associated sensory neuropathy found that participants receiving gabapentin were more likely to report somnolence (12/15 for gabapentin and 2/11 for placebo; *p* = 0.006), dizziness (9/15 for gabapentin and 5/11 for placebo; *p* = 0.305), and gait disturbance (7/15 for gabapentin and 3/11 for placebo; *p* = 0.357) (Hahn et al., 2004).

Notably, we observed a dose–response relationship for adverse events consistent with findings of a meta‐analysis examining gabapentin safety among patients with postherpetic neuralgia (Wang and Zhu, 2017). This study reported increased risk of adverse outcomes starting at gabapentin doses of ≥ 1,800 mg/d, whereas we demonstrated increased risk at lower doses of ≥ 600 mg/d. These findings are particularly relevant when considering the use of gabapentin to treat AUD, as current evidence suggests greater impact of gabapentin on reducing alcohol consumption at higher doses of ≥ 1,500 mg/d (Mason et al., 2014; Rentsch et al., 2019). However, it is important to note that gabapentin has been shown to improve AUD outcomes even at doses as low as 900 mg/d (Mason et al., 2014), which may reduce the risk of adverse consequences. Our finding of continued gabapentin prescribing at low or subtherapeutic doses despite the apparent risk of adverse events may reflect a desire among clinicians to prescribe nonopioid medications for pain (Goodman and Brett, 2017).

This research differs from recent safety studies of gabapentin use in a number of important ways. First, we evaluated the association of gabapentin with neurologic adverse events in a real‐world setting among patients who were prescribed gabapentin for any indication. Second, we addressed methodological challenges inherent to observational study designs by applying uniform exclusion criteria for exposed and unexposed patients, evaluating incident exposures, setting an index date for exposed and unexposed patients, and using propensity score matching to account for confounding by indication. Finally, our study included a large sample size of approximately 140,000 gabapentin‐exposed patients, which to our knowledge is the largest study to date examining gabapentin safety in a real‐world setting.

There are limitations to our work. Due to characteristics of individuals who access care in the VA healthcare system, our sample was enriched with men and patients with multiple medical comorbidities, which may not generalize to other clinical settings. Some of our analyses lacked adequate power due to small samples in certain patient subgroups, including patients with HIV infection. We were also unable to obtain proxy measures to capture adverse outcomes that were not diagnosed using ICD‐9 codes, which may underestimate the rate of adverse events. Only one‐third of the gabapentin‐exposed patients in our cohort were propensity‐score matched, although there was no significant difference of HCV, HIV, or AUD prevalence between those who did and did not match. Our findings may not generalize to those with stronger indications for gabapentin. Despite these limitations, we believe our findings provide novel information on the safety of gabapentin use and highlight the risk of falls, fractures, and altered mental status among patients from a large national integrated healthcare system.

This work has important implications for researchers and clinicians. We used real‐world data to demonstrate that gabapentin is associated with increased risk of falls, fractures, and altered mental status at doses ≥ 600 mg/d, which should be carefully considered by clinicians. The widespread prescribing of gabapentin for various conditions, which contrasts with the limited use of FDA‐approved medications for AUD, suggests that clinicians are familiar with gabapentin and may feel more comfortable prescribing it for AUD treatment. Such comfort with prescribing gabapentin paired with growing evidence to support its efficacy in treating AUD (Anton et al., 2020; Kranzler et al., 2019; Mason et al., 2014; Pani et al., 2014) may help expand the number of individuals with AUD receiving effective medication treatment. However, these possible benefits must be weighed against potential risks. There is evidence suggesting nonmedical use of gabapentin to achieve euphoric effects, particularly in individuals with substance use disorders (Peckham et al., 2017; Smith et al., 2016). Although gabapentin misuse has been reported primarily in individuals with opioid and polysubstance use rather than in those with AUD alone, clinicians should consider monitoring for medication misuse and diversion when prescribing gabapentin. Although we did not find an excess risk of adverse events among gabapentin‐exposed patients with current AUD, our findings indicate that these patients are predisposed to falls, fractures, and altered mental status that may reflect complications of acute and chronic alcohol use, such as intoxicating effects, advanced liver disease, and peripheral neuropathy. More research is needed to clarify predisposing risk factors and drug interactions that may increase these neurologic adverse events in patients with AUD to guide risk stratification and to examine gabapentin safety at higher doses associated with improved AUD outcomes.

## Sources of Support

This work was supported by National Institute on Alcohol Abuse and Alcoholism [R01‐AA023733, U24‐AA020794, U01‐AA020790, U10‐AA013566].

## Conflicts of Interest

The authors declare no conflict of interest.

## Supporting information


**Table S1** Distribution of baseline characteristics in gabapentin exposed patients who did and did not propensity score (PS) match.
