# Supplementary material for: Safety of Gabapentin Prescribed for Any Indication in a Large Clinical Cohort of 571,718 US Veterans with and without Alcohol Use Disorder
Source: Alcohol Clin Exp Res. 2020 Jul 28;44(9):1807–15. doi: 10.1111/acer.14408 (PMC7540277; doi:10.1111/acer.14408)
Supplement: Supplementary file 1 — Table S1 Distribution of baseline characteristics in gabapentin exposed patients who did and did not propensity score (PS) match. [file ACER-44-1807-s001.docx]

| **Table S1.** Distribution of baseline characteristics in gabapentin exposed patients who did and did not propensity score (PS) match | | | | |
| --- | --- | --- | --- | --- |
|  | **Did not PS-match** |  | **PS-matched** |  |
| **Characteristic** | **n=291,610** |  | **n=140,310** | **Std. Diff** |
| Propensity for exposure, median (IQR) | 0.11 (0.03-0.27) |  | 0.07 (0.03-0.13) | 0.57 |
| Age |  |  |  |  |
| *<60* | 178,348 (61.2) |  | 88,980 (63.4) | 0.05 |
| ≥*60* | 113,262 (38.8) |  | 51,330 (36.6) |  |
| Race/ethnicity |  |  |  |  |
| *White* | 205,102 (70.3) |  | 97,513 (69.5) | 0.04 |
| *Black* | 51,318 (17.6) |  | 24,815 (17.7) |  |
| *Hispanic* | 15,655 (5.4) |  | 7,512 (5.4) |  |
| *Other* | 9,064 (3.1) |  | 4,454 (3.2) |  |
| *Missing* | 10,471 (3.6) |  | 6,016 (4.3) |  |
| Male sex | 270,930 (92.9) |  | 131,007 (93.4) | 0.02 |
| HCV+ | 27,480 (9.4) |  | 12,384 (8.8) | 0.02 |
| HIV+ | 2,812 (1.0) |  | 1,381 (1.0) | <0.01 |
| AUD |  |  |  |  |
| *Never* | 225,447 (77.3) |  | 111,937 (79.8) | 0.06 |
| *Lifetime* | 30,075 (10.3) |  | 13,336 (9.5) |  |
| *Current* | 36,088 (12.4) |  | 15,037 (10.7) |  |
| Any hospitalization | 47,560 (16.3) |  | 20,494 (14.6) | 0.05 |
| Conditions |  |  |  |  |
| *Seizure* | 7,719 (2.7) |  | 3,166 (2.3) | 0.03 |
| *Diabetes* | 114,068 (39.1) |  | 51,017 (36.4) | 0.06 |
| *Anxiety* | 58,607 (20.1) |  | 24,277 (17.3) | 0.07 |
| *Neuropathic pain* | 82,640 (28.3) |  | 28,524 (20.3) | 0.19 |
| *Any chronic pain* | 272,922 (93.6) |  | 129,072 (92.0) | 0.06 |
| Other prescription |  |  |  |  |
| *Benzodiazepines* | 43,229 (14.8) |  | 17,786 (12.7) | 0.06 |
| *Opioid* | 87,715 (30.1) |  | 32,163 (22.9) | 0.16 |
| *Antidepressant* | 38,105 (13.1) |  | 13,697 (9.8) | 0.10 |
| *NSAID* | 144,390 (49.5) |  | 62,935 (44.9) | 0.09 |
| *Muscle relaxant* | 51,871 (17.8) |  | 17,755 (12.7) | 0.14 |
| *Anticonvulsant* | 8,756 (3.0) |  | 3,631 (2.6) | 0.03 |
| Notes: all statistics reported as n (%), unless otherwise noted | | | | |
| Abbreviations: PS - propensity score; IQR - interquartile range; HIV - human immunodeficiency virus; HCV - hepatitis C virus; AUD - alcohol use disorder; NSAID - non-steroidal anti-inflammatory drug | | | | |
